# Unsupervised Machine Learning Identifies Quantifiable Patterns of Visual Field Loss in Idiopathic Intracranial Hypertension

**DOI:** 10.1167/tvst.10.9.37

**Published:** 2021-08-30

**Authors:** Hiten Doshi, Elena Solli, Tobias Elze, Louis R. Pasquale, Michael Wall, Mark J. Kupersmith

**Affiliations:** 1 Albert Einstein College of Medicine, Montefiore Medical Center, Bronx, NY, USA; 2 Neurology, Icahn School of Medicine at Mount Sinai, New York, NY, USA; 3 Ophthalmology, Icahn School of Medicine at Mount Sinai, New York, NY, USA; 4 Schepens Eye Research Institute, Harvard Medical School, Boston, MA, USA; 5 Department of Neurology and Ophthalmology and Visual Sciences, University of Iowa, Iowa City, IA, USA

**Keywords:** visual field, archetypal analysis, IIH, papilledema, unsupervised learning

## Abstract

**Purpose:**

Archetypal analysis, a form of unsupervised machine learning, identifies archetypal patterns within a visual field (VF) dataset such that any VF is described as a weighted sum of its archetypes (ATs) and has been used to quantify VF defects in glaucoma. We applied archetypal analysis to VFs affected by nonglaucomatous optic neuropathy caused by idiopathic intracranial hypertension (IIH).

**Methods:**

We created an AT model from 2862 VFs prospectively collected from 330 eyes in the IIH Treatment Trial (IIHTT). We compared baseline IIH AT patterns with their descriptive VF classifications from the IIHTT.

**Results:**

The optimum IIH AT model yielded 14 ATs resembling VF patterns reported in the IIHTT. Baseline VFs contained four or fewer meaningful ATs in 147 (89%) of study eyes. AT2 (mild general VF depression pattern) demonstrated the greatest number of study eyes with meaningful AT weight at baseline (n = 114), followed by AT1 (n = 91). Other ATs captured patterns of blind spot enlargement, hemianopia, arcuate, nasal defects, and more nonspecific patterns of general VF depression. Of all ATs, AT1 (normal pattern) had the strongest correlation with mean deviation (*r* = 0.69, *P* < 0.001). For 65 of the 93 VFs with a dominant AT, this AT matched the expert classification.

**Conclusions:**

Archetypal analysis identifies quantifiable, archetypal VF defects that resemble those commonly seen in IIH.

**Translational Relevance:**

Archetypal analysis provides a quantitative, objective method of measuring and monitoring disease-specific regional VF defects in IIH.

## Introduction

Visual field (VF) testing is used to diagnose and monitor most optic neuropathies. Changes in threshold perimetry detected through trend or event-based analyses are often used to determine change in visual function. Many commonly used methods, including assessing change in mean deviation (MD), however, do not provide details of regional VF defects.^1^^,^^2^ Experts categorize specific patterns of VF loss, but clinical interpretation is descriptive and qualitative. Thus longitudinal analyses are subjective, leading to conflicting opinions. Supervised learning algorithms have been used in glaucoma to monitor disease-specific deficits, but the results of these algorithms frequently disagree with one another.^3^^,^^4^ Each applies ad hoc rules depending on the stage of disease, and no universally accepted approach exists.^5^ Furthermore, subtle patterns of regional VF loss can be missed, some disease-specific deficits are not tracked, and the algorithms are often arbitrarily weighted to preferred regions such as the central field.

A new approach to VF analysis uses archetypal analysis (AA), an unsupervised machine learning technique. Previously, AA has been used in a variety of settings pertaining to physics and engineering, such as the analysis of air pollution data, and the assessment of the head and face for military mask fitting.^6^^,^^7^ AA processes a heterogeneous dataset and identifies representative patterns along the outer edges of the data space (the edges of which are known collectively as the “convex hull”). These representative patterns referred to as “archetypes” (ATs), and together they comprise a mathematically determined model such that any observation within the dataset can be described as a weighted combination of these ATs.^6^^–^^8^ When AA is applied to a dataset of VFs taken from eyes affected by a specific disease, AA can identify the distinct types of VF patterns seen in this disease (as they are represented within the dataset) as ATs.^8^^,^^9^ AA has already successfully identified patterns of VF loss in glaucoma that were very similar to expert-determined patterns reported in the Ocular Hypertension Treatment Study.^4^^,^^8^^,^^10^^–^^13^ Once an archetypal model (with each AT representing a different type of VF pattern or regional defect) is generated for a dataset of VFs, any VF can be decomposed into its specific component ATs, each with a relative percent weight.^8^^,^^10^^,^^13^ A given VF can thus be described as a weighted sum of all ATs within the model, with higher percent weights attributed to those ATs which are most highly represented within the given VF pattern. AA therefore provides quantification of specific patterns of regional VF loss.

To date, AA has not been applied to evaluate nonglaucomatous optic neuropathies, where functional improvement can be achieved with therapy unlike in glaucoma, where the goal is prevention of further loss of function.^14^^–^^18^ Idiopathic intracranial hypertension (IIH) is one such disorder, where visual dysfunction, recovery, and response to therapy are common, but visual deficits vary widely.^14^^,^^17^ AA has the potential to provide quantifiable metrics that can be used to monitor therapy and measure improvement or worsening in IIH VF patterns. This could reduce dependence on subjective clinician VF interpretation and improve the accuracy and uniformity of VF analysis. Here, we apply AA to the VF dataset from the Idiopathic Intracranial Hypertension Treatment Trial (IIHTT).^17^ We assessed whether AA can independently reveal patterns of VF loss in IIH that resemble known VF defects typically seen in IIH and quantify these patterns of VF loss.

## Methods

This study was approved by the Institutional Review Board of the Icahn School of Medicine at Mount Sinai. The IIHTT followed the tenets of the Declaration of Helsinki; informed consent was obtained from the subjects after explanation of the nature and possible consequences of the study; the research was approved by the institutional human experimentation committee or institutional review board; and a Data Safety and Monitoring Committee was in place to monitor the ethical conduct of the study and the accumulation of data for evidence of adverse and beneficial treatment effects.

### Datasets

This study used data from the National Eye Institute–sponsored IIHTT, collected from 165 IIH subjects (330 eyes) with mild VF loss (MD of −2 to −7 dB required as inclusion criteria) who were randomized to a supervised weight loss diet with acetazolamide or the same diet with placebo.^17^ The mean age of participants was 29 years (range 18–52 years), and 97.6% of participants were female. Mean body mass index was 39.9 (range 24.9 to 71.2), and mean CSF opening pressure on lumbar puncture was 343.5 mm H_2_0 (range 210–670 mm H_2_O). Papilledema was graded according to the Frisén scale, with a grade of 2 being the most common (at least grade 1 required for study entry).^19^ The VFs were conducted by trained, certified technicians with quality control by an expert visual field reading center (VFRC).^20^^,^^21^ The eye with worse MD at presentation was labeled the “study eye,” and the other eye was considered the “non-study eye.” For study eyes at baseline, the average MD was −3.5 ± 1.1 dB in study eyes and −2.3 ± 1.1 dB in non-study eyes (± standard deviation). Visual acuity was measured via the Early Treatment Diabetic Retinopathy Study (ETDRS) method, with a score of 85 letters equal to 20/20. The visual acuity was 20/20 or better in 70.9% of study eyes and 77.0% of non-study eyes at baseline. Subjects had reliable VFs performed on Humphrey Field Analyzer 24-2 SITA Standard testing at six visits according to the following schedule: two VFs were performed for each eye at baseline, one VF at each additional visit at one, two, three, four, and five and two VFs at six months. The MD and pattern standard deviation (PSD) values (decibels [dB]) were reported for each VF at each visit for each eye.

Additional VFs were performed if the results were suggestive of treatment failure (see below) or were considered unreliable by the IIHTT Visual Field Reading Center. All VF tests required adequate gaze tracking and reliability standards of fixation loss errors <33% and false-positive errors <15% to be included. At any time during the IIHTT, treatment failure was determined if the MD decreased by ≥2 dB from presentation when baseline MD was ≤−3.5 dB or if MD decreased by ≥3 dB from presentation when baseline MD was between −3.5 and −7 dB, which was confirmed on repeat VF testing. Performance failures were identified based on VF worsening that could not be reproduced on subsequent VF exams, indicating that this apparent worsening could be explained by lack of effort, attention, or concentration rather than true clinical worsening. The IIHTT Visual Field Reading Center and study leadership identified seven study subjects as treatment failures and 35 subjects as performance failures.^20^

This IIHTT dataset contained a total of 2933 VFs taken over multiple time points, from baseline to one year. VFs marked as “unreliable” were removed, leaving 2862 VFs from both eyes remaining that were used to generate the IIH ATs. For our further analyses, we excluded visits occurring after six months, defining an “outcome visit” as the six-month visit for all eyes. If the subject was a treatment failure, we substituted the VF taken at the treatment failure visit for all other visits through six months. Because subjects were treated with a variety of interventions including surgery, which could alter the outcome, we excluded VFs taken at any other visit occurring after confirmation of treatment failure. For performance failures, only the reliable confirmatory VFs were included. For all eyes, if multiple reliable VFs existed for a single time point, we used the average TD values at each point location for that time. This process left 1683 VFs remaining (839 from study eyes and 844 from non-study eyes), which were used in the remainder of the analysis.

A separate dataset of 568 VFs, collected from 61 normal eyes of 61 subjects with repeated 24-2 VF tests performed at the University of Iowa was used as a control group to establish the normal fluctuation of IIH AT weights (see below for an explanation of AT weights) among healthy eyes. The mean age of participants was 61.22 years (range 42–79 years), and 63.3% were women. Participants were included if they met the following criteria: (1) no history of eye disease except refractive error (no more optical correction than 5 Diopter of sphere or 3 Diopter of cylinder), (2) no history of diabetes mellitus or systemic arterial hypertension, (3) a normal ophthalmologic examination including 20/25 or better Snellen acuity, (4) good fixation (losses < 20%) by gaze tracking or perimetrist observation, and (5) false-positive and false-negative rates of less than 10% on perimetry.^22^

### Archetypal Analysis

**Figure 1.**
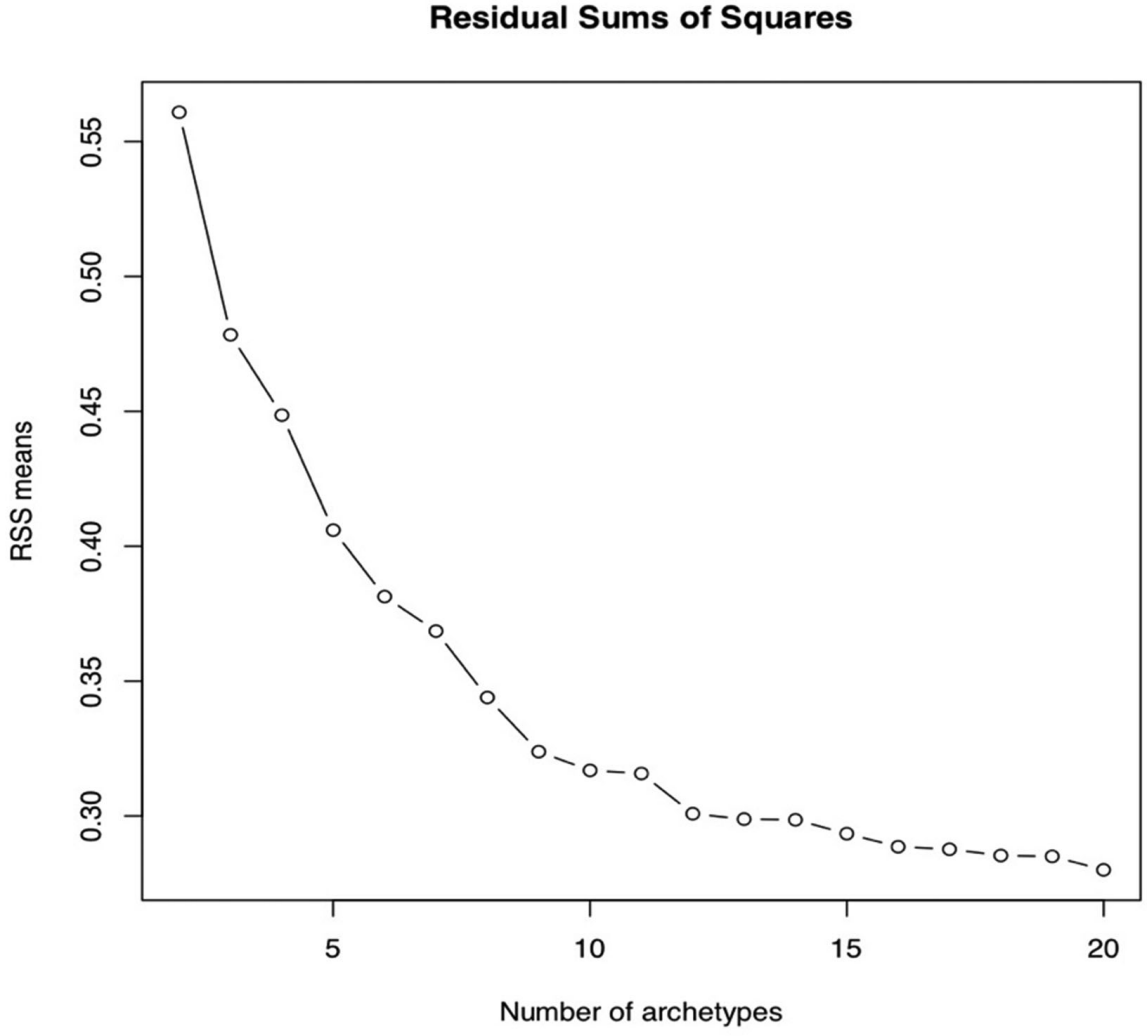
RSS plot created for selection of idiopathic intracranial hypertension AT model. The final number of ATs for our 14-AT model was selected based on the point at which the curve begins to flatten in order to avoid overfitting.

We used the open-source software package “archetypes” within the R statistical programming environment (R Development Core Team 2008) to perform AA on 2862 VFs from the IIHTT. Total deviation (TD) values (extracted from these 24-2 VFs) from all visits occurring up to one year were included as input data for the generation of our AT model. We used 10-fold cross validation to select the number of ATs (such that all data was divided into 10 subsets, with each being used as the testing set once, and the others serving as the training set) for our model. Using TD values from our dataset of IIH VFs, we calculated the residual sum of squares (RSS) for models using two to 20 archetypes. We plotted the number of archetypes against their RSS values and selected the final model based on the value where the RSS curve flattened to avoid overfitting (Fig. 1)*.* The weights for all ATs within the IIH model for each VF summed to 100%. For comparison, we also applied AA to our dataset of 568 healthy control VFs to create a set of control ATs, and to establish normal fluctuation in ON weighting coefficients among healthy eyes over time.

### Defining Threshold Value for a Meaningful Weight and Weight Change

We defined the minimum AT weighting coefficient necessary to represent a minimum meaningful AT weight, as well as the minimum AT weight change necessary to represent meaningful change in vision as (1) a value greater than the upper limit of the 95% confidence interval (CI) for any of the abnormal IIH ATs weights calculated for control eyes, and (2) a value greater than the upper limit of the 95% CI for any IIH AT weight changes calculated for control eyes. To accomplish this, we decomposed all control VFs into the IIH ATs and calculated both the mean weight of each IIH AT for control eyes and the mean weight change of each IIH AT for control eyes, averaging VFs performed over one year. The greatest upper limit of the 95% CI for any abnormal IIH AT weight among control eyes was >5% (excluding AT1, a normal AT), and the greatest upper limit of the 95% CI for any IIH AT weight *change* among control eyes was 7%. Because all control VFs were classified as normal, AT weight changes that fell within confidence intervals were considered to represent normal fluctuation, not reflective of meaningful weight change. We also factored in the probability that 1/14 ATs could show >7% weight. We chose a very conservative value of ≥9% to represent a meaningful weight or weight change for any IIH AT.

### Visual Field Decomposition, and Statistical Analyses

After selection of a 14-AT model for our IIH VF dataset, we decomposed all baseline and one-month IIH VFs into their component ATs. We tabulated the number of study eyes and non-study eyes with relevant AT weight (≥9%) at baseline. The χ^2^ analysis was used to assess for significant difference between the treatment groups with regard to frequency of study eyes with any AT weight ≥9% at baseline. Wilcoxon tests were used to assess for any significant difference in mean AT weights between individual treatment groups for study eyes at baseline. Spearman's correlation was used to test for any significant relationship between each individual AT weight and MD, PSD, visual acuity, and Frisén grade among study eyes. Spearman's correlation was also used to evaluate the relationship between baseline study eye AT Sum scores (to be described), MD, and PSD.

### Development of Composite Baseline AT Sum Score

We created a composite score (AT Sum) that could represent a measure of VF dysfunction and reflect peripheral and central VF deficits at a single time point. To calculate this score, we assigned a positive sign to all weighting coefficients for normal or less abnormal ATs (ATs 1, 11, 7, 2, 8, 4 and 12; those with higher average TD values), and a negative sign to the weighting coefficients for the more abnormal ATs (ATs 5, 3, 6, 9, 10, 13, and 14; those with lower average TD values). For an individual VF, the AT sum score is equal to the sum of these 14 adjusted weighting coefficients, with lower values representing worse VF function, and higher values representing superior VF function. In contrast to MD, the AT sum is representative of all abnormal regional deficits and not dominated by the central VF points.

### Comparison of ATs to Known IIH VF Patterns

Two authors (MK and MW) categorized the 14 IIH ATs using the same approach used by the VFRC for classifying the IIHTT visual fields.^20^^,^^21^ Although masked to the IIHTT expert categorizations, we applied these classifications to the dominant AT (≥50% weight) of the baseline visual fields for all IIHTT eyes. We compared these dominant ATs to the expert classifications already determined by the VFRC, noting if the results were the same, partially the same (containing similar clinically relevant features), or different.

## Results

### Development of a 14-AT Model for IIH

**Figure 2.**
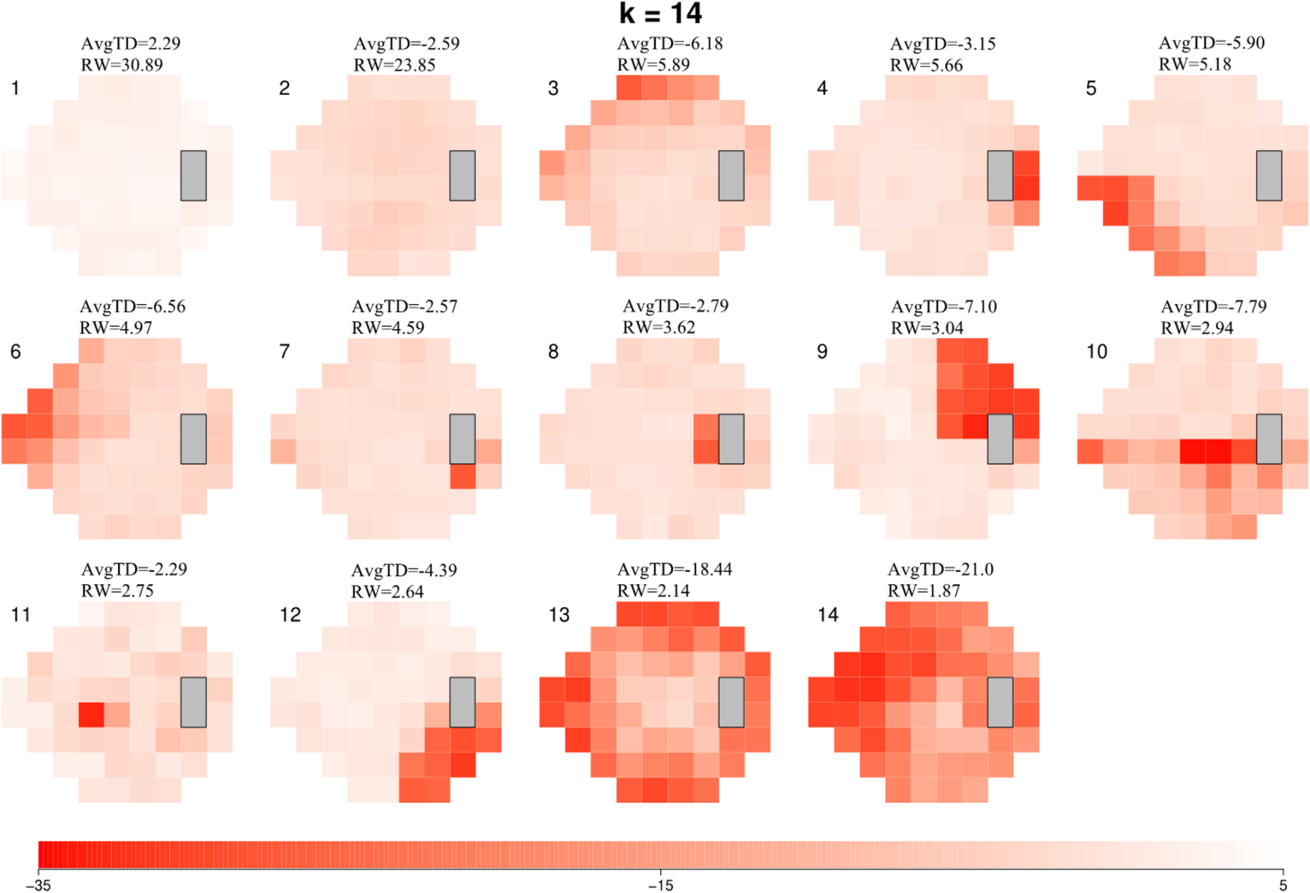
These are the 14 ATs we derived from the IIHTT dataset (including all eyes and examination dates up to one year). These ATs are shown in order of relative weight, representing their frequency within the dataset. The scale at the bottom denotes the TD values associated with each of the varying shades of red within each AT (range −35 to 5.0 dB). Each AT is displayed along with its corresponding average TD value (in dB) and relative weight (RW) within the dataset. AvgTD, average total deviation.

The optimal AT model created from the IIHTT VF dataset (using the RSS criteria described in methods) (Fig. 1) had 14 archetypes. The IIH model showed a wide range of ATs, each with a specific average TD value, and weighting coefficient relative to the frequency of each AT within the dataset (Fig. 2)*.* AT1, a normal AT, had the highest relative weight at 30.89%. This was followed by AT2, a mild general depression pattern AT, with a relative weight of 23.85%. Based on this 14-AT model, all study eye VFs were decomposed into their component IIH ATs; with each VF represented by 14 AT weighting coefficients, summing to 100%.

In addition, we created a 12-AT model for the dataset of 568 VFs taken from healthy “control” eyes with normal vision (Supplementary Fig. S1). Control ATs were distinct from IIH-specific ATs. The majority of control ATs were consistent with normal vision. As such for the IIH ATs, AT1 was the most frequent AT (highest relative weight, 14.9%) and also had the highest average TD value (3.25 dB) in control eye visual fields.

### Baseline Archetype Weights

**Figure 3.**
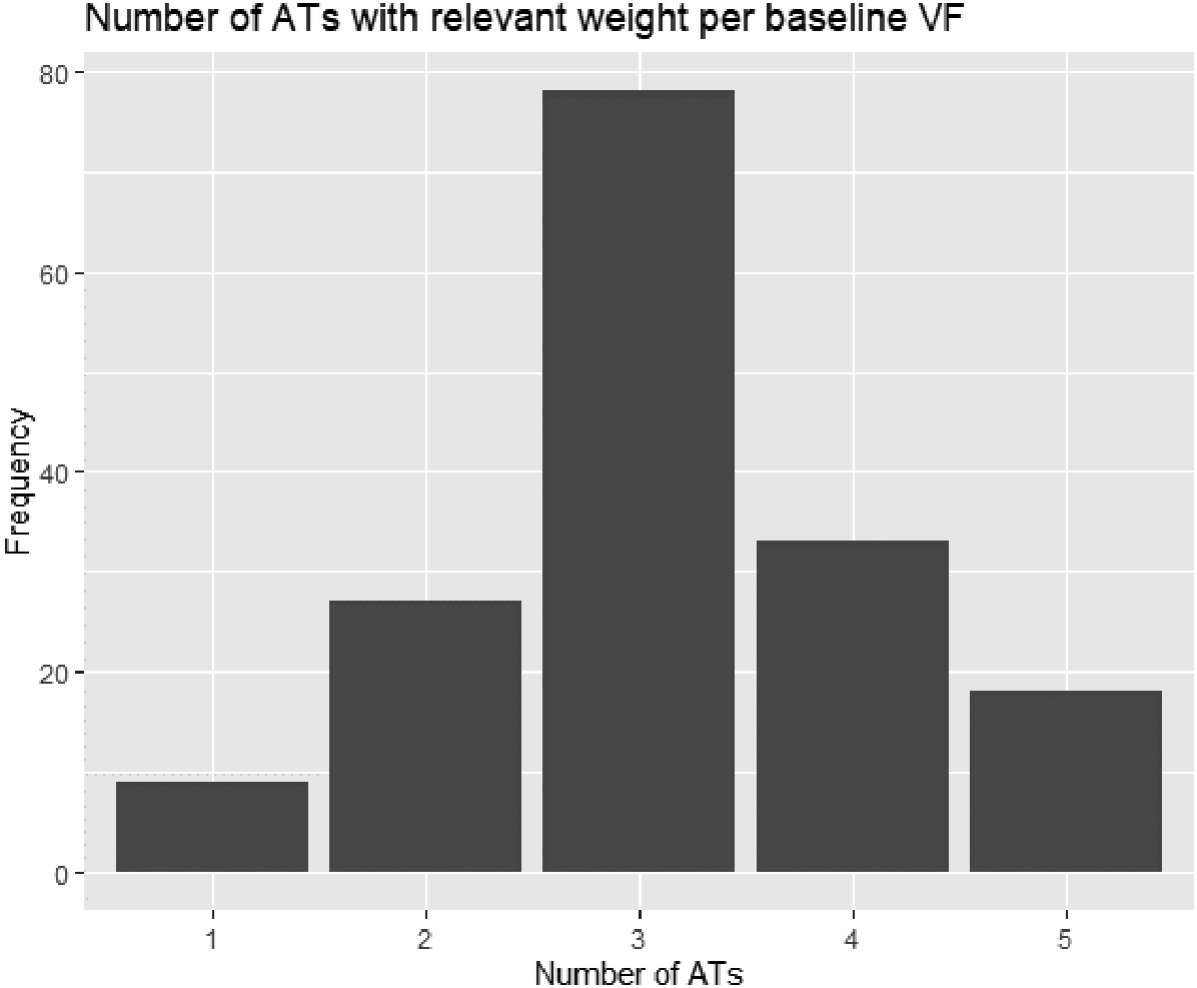
Frequency of baseline study eye visual fields containing listed number of archetypes with meaningful weight (≥ 9%).

**Figure 4.**
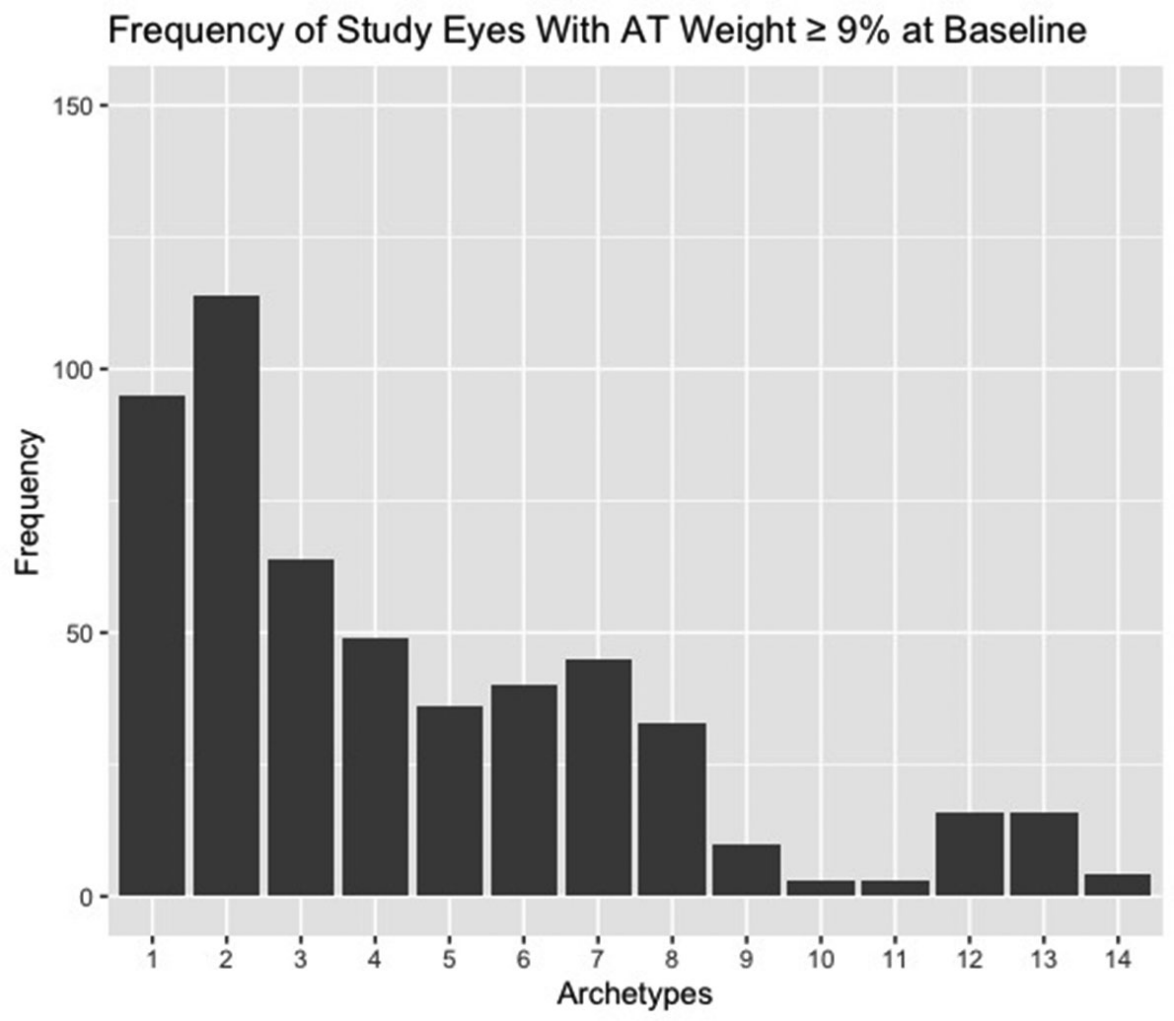
Frequency of study eyes with archetype weight ≥ 9% at baseline (for each archetype as listed).

The average number of ATs with meaningful weighting contained within each baseline VF was 3.2 ± 1. Eighty-nine percent (N = 147) of baseline VFs could be decomposed into four ATs or less, and none contained more than 5 ATs (Fig. 3). The frequency of study eyes (N = 165) with meaningful weight (≥ 9%) for each AT at baseline (Fig. 4) shows that the ATs with the poorest average TD values (except AT11 with mildly abnormal TD) were less common at baseline (in keeping with the mild average MD values seen at study entry)***.*** Non-study eyes (N=165) showed a similar distribution of baseline AT weights (Supplementary Fig. S2). AT2 demonstrated the greatest number of study eyes with meaningful weighting at baseline (N = 114), followed by AT1 (N = 95). The mean weights at baseline for all AT for all study eyes are summarized in Supplementary Table S1. AT2 showed the highest mean weight for any baseline AT (23.9%; 95% CI 20.9%–26.9%), followed by AT1 at 14.7% (95% CI 12.6%–16.7%).

### Correlation of AT Weights With Other Clinical Features At Baseline

**Figure 5.**
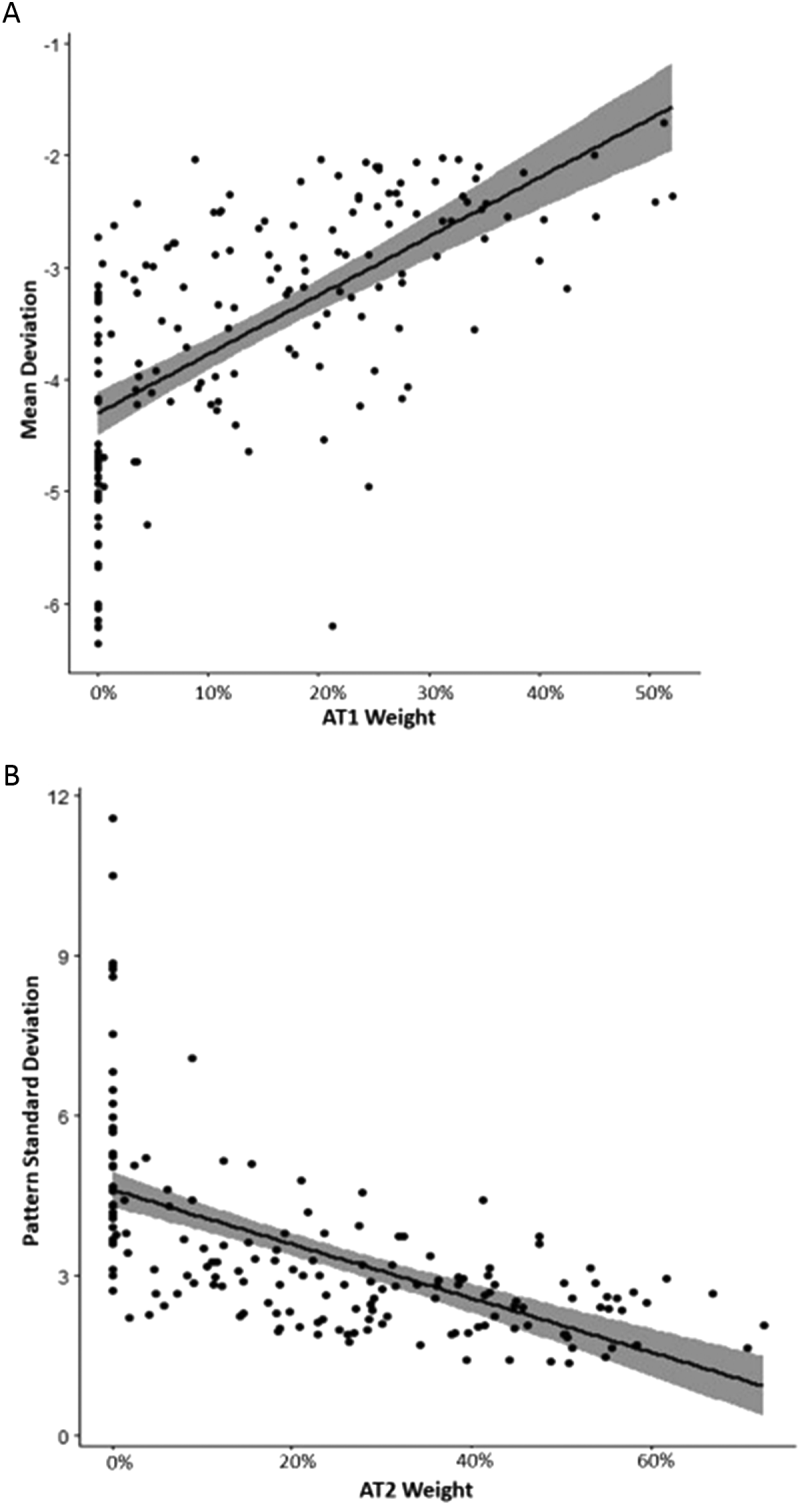
(**A**) Spearman correlation between AT1 weight and mean deviation (dB) at baseline (*r* = 0.69, *P* < 0.001) for study eyes. (**B**) Spearman correlation between AT2 weight and pattern standard deviation (dB) at baseline (*r* = −0.68, *P* < 0.001) for study eyes. AT1, archetype 1; AT2, archetype 2.

**Figure 6.**
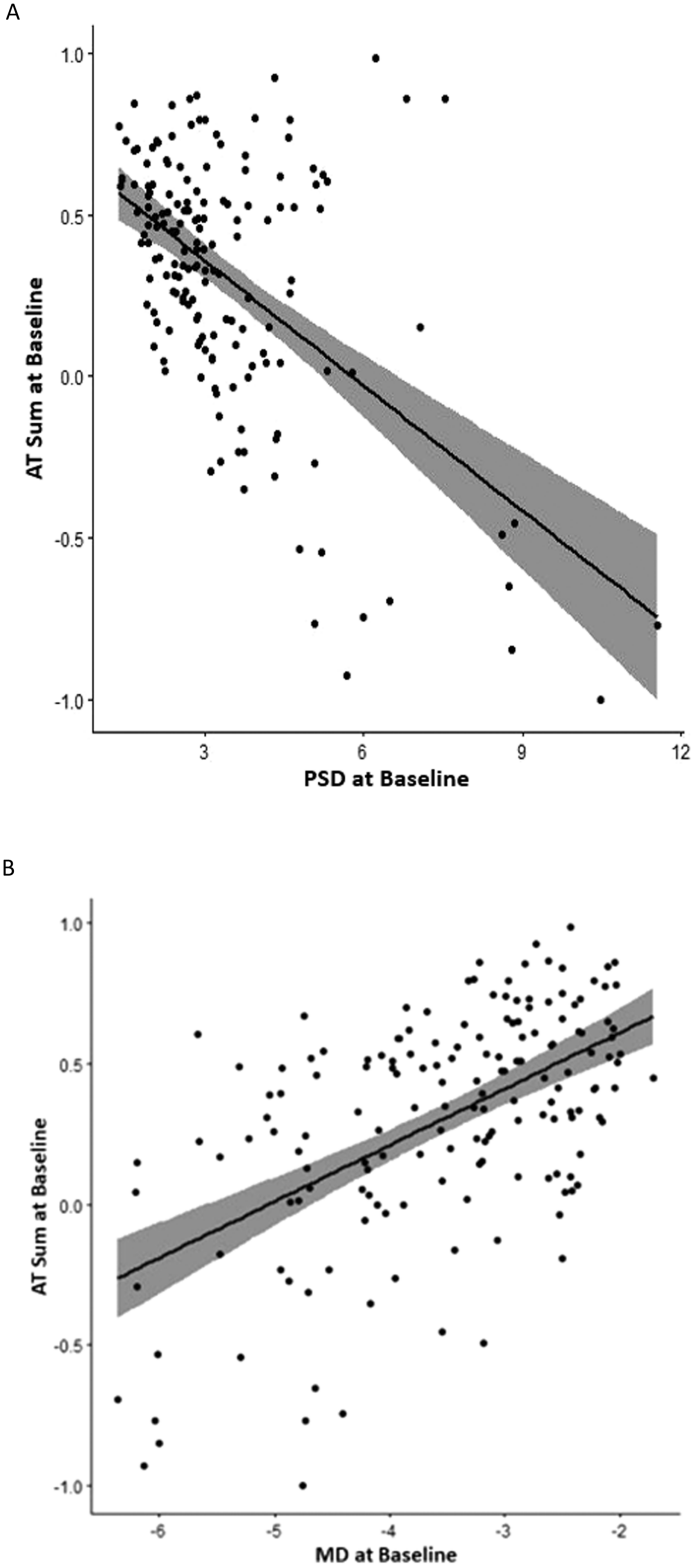
(**A**) Spearman correlation between AT Sum and pattern standard deviation (dB) at baseline (*r* = −0.37, *P* < 0.001) for study eyes. (**B**) Spearman correlation between AT Sum and mean deviation (dB) at baseline (*r* = 0.48, *P* < 0.001) for study eyes.

At baseline, AT1 weight demonstrated the strongest overall correlation with MD (*r* = 0.69, *P* < 0.001; Fig. 5A**)**, and AT2 demonstrated the strongest correlation with PSD (*r* = −0.68, *P* < 0.001; Fig. 5B). AT1 and PSD were mildly correlated, (*r* = −0.34, *P* < 0.001), and AT2 was not correlated with MD. MD and PSD correlated more modestly with select additional baseline AT weights (Supplementary Tables S2 and S3). Baseline AT Sum scores for all study eyes correlated mildly with PSD at baseline (*r* = −0.37, *P* < 0.001; Fig. 6A), and moderately with MD at baseline (*r* = 0.48, *P* < 0.001; Fig. 6B). There was no significant correlation between any individual AT weight and visual acuity at baseline. Reflecting the large blind spot in the visual field, both AT7 and AT8 displayed a mild correlation with Frisén grade at baseline (AT7: *r* = 0.37, *P* < 0.001; AT8: *r* = 0.37, *P* < 0.001). Select additional ATs showed modest correlations with Frisén grade; however, none correlated more strongly than AT7 or AT8 (Supplementary Table S4).

### Comparison of ATs to Known IIH VF Patterns

The 14-archetype IIH model showed a wide variety of visual field patterns similar to those reported in the IIHTT by VF experts including blind spot enlargement, arcuate and partial arcuate defect, quadrantanopia, generalized VF depression, and normal VF.^21^ We analyzed 93 VFs that met criteria for having a dominant AT (weight ≥ 50%) at baseline. The dominant AT classification for 39 (42%) of these VFs was an exact match to the expert category, and 26 (28%) were a partial match. Thus, for 70% of these 93 VFs, the dominant AT displayed at least a partial match with the expert classification (Supplementary Table S5). Differences in classification occurred in 28 visual fields, 23 of which were classified by AT2 (mild widespread loss), whereas the expert classification for the actual VF was “superior and inferior partial arcuate defect.”

**Figure 7.**
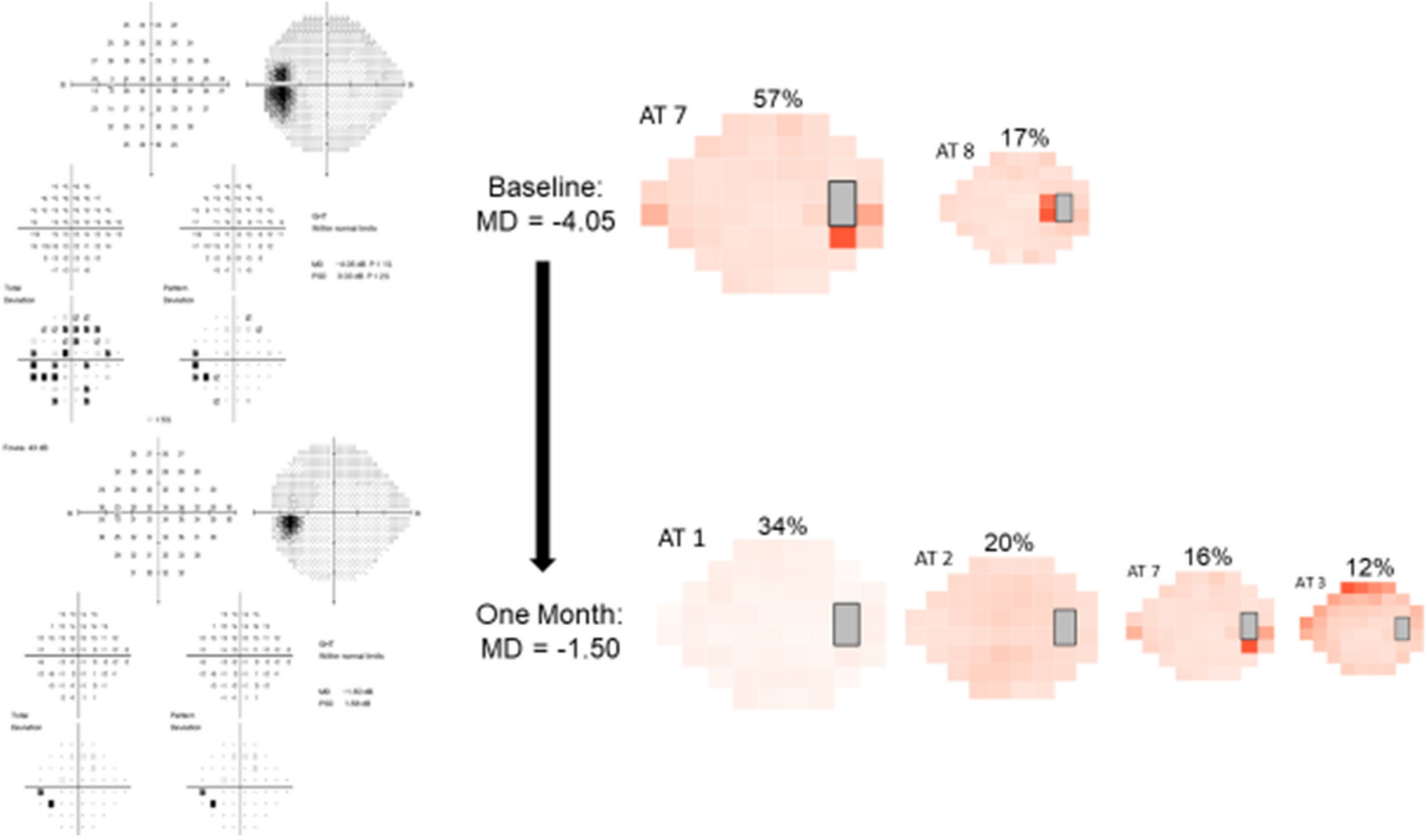
Example of progression of VF and AT weight changes between baseline and one month in one study eye. At baseline, this eye's VF was characterized by a nerve fiber bundle-type defect with enlarged blind spot (the most common type seen in the idiopathic intracranial hypertension treatment trial). Broken down into ATs, this baseline VF was dominated by AT7 at a weight of 57%, with a partial contribution from AT8. After receiving acetazolamide, the VF began to improve at one month. Although a small contribution from AT2 and AT3 remain, we observe a decrease in AT7 weight, as well as a new increase in AT1 (representing a normal VF) weight. Note ATs with weight < 9% not shown (see methods).

AA can show minor changes in the VF between visits. For example, Figure 7 demonstrates the progression of VF and AT weight changes between baseline and one month for one study eye. At baseline, the VF is characterized by a nerve fiber bundle–type defect with enlarged blind spot (the most common type seen in the IIHTT). Broken down into ATs, this baseline VF was dominated by AT7 at a weight of 57%, with a partial contribution from AT8. At one month, the VF shows improvement after treatment with acetazolamide. While a small contribution from AT2 and AT3 remain, we observe a decrease in AT7 weight, as well as a new increase in AT1 (representing a normal VF) weight.

**Figure 8.**
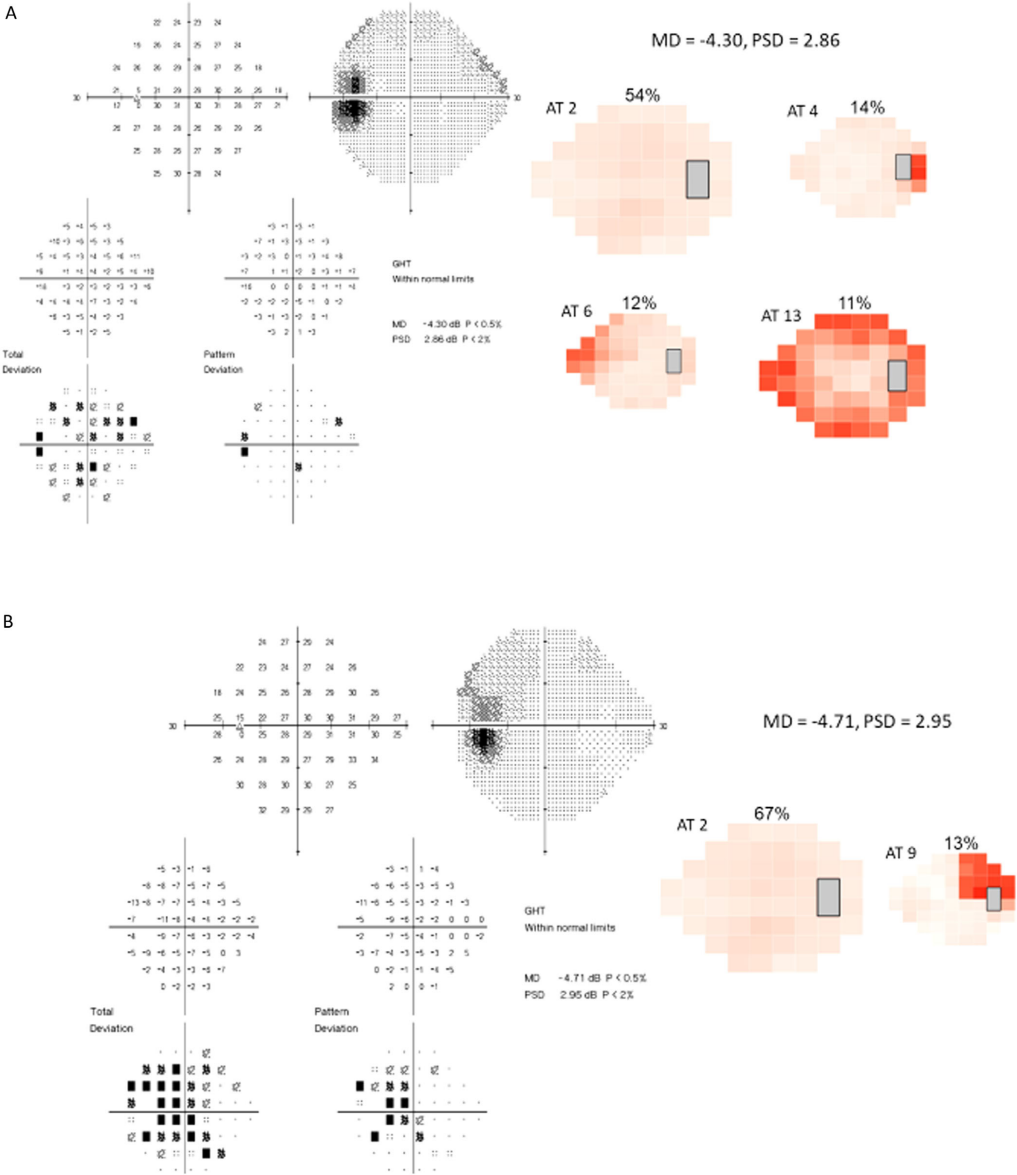
(**A** and **B**) Baseline visual fields from two different study eyes with similar MD and PSD values. Although MD and PSD are comparable in both fields, the composition of AT weights remains noticeably different between the two, with the eye in A showing more abnormal ATs.

The two study eyes in Figure 8A and 8B illustrate that even when VFs from two different eyes have comparable MD and PSD values, the decomposed ATs for individual eyes may be distinct each with specific regional deficits. Although AT2 weight is high in both baseline fields, the remaining ATs composing each VF differ; the eye in Figure 8A, includes AT4, AT6, and AT13, and the eye in Figure 8B, includes AT9.

## Discussion

Through archetypal analysis (AA), we identified quantifiable, disease-specific patterns or archetypes of VF deficits in eyes affected by IIH. These patterns are similar to the descriptive patterns previously reported in the IIHTT, however AA revealed additional regional deficits not always seen by inspection. AA is a form of unsupervised machine learning that is capable of revealing component patterns within a variety of heterogeneous datasets.^4^^,^^6^^–^^8^^,^^10^^,^^11^ AA has previously been studied in glaucoma, and has successfully identified patterns of glaucomatous VF loss that closely resemble the expert classifications of VF defects described in the Ocular Hypertension Treatment Trial.^4^^,^^8^^,^^10^^–^^13^^,^^23^ This study is the first reported application of AA to VF analysis in eyes affected by a nonglaucomatous optic neuropathy.

Applying AA to a large dataset of VFs collected during the IIHTT, we created a 14-AT model for IIH, such that any individual VF within the dataset could be described by a weighted combination of these quantifiable archetypal patterns. Interestingly, although unsupervised machine learning offers the possibility of identifying previously unknown patterns within a dataset, practically all ATs derived from this dataset resembled known VF patterns seen in IIH, although some were not obvious without AA decomposition. In addition, in individual VFs with dominant ATs (> 50% weight), VF patterns associated with each dominant AT were consistent with original descriptive expert classifications reported for the IIHTT. VF patterns seen in IIH have been described as localized nerve fiber bundle defects (including arcuate), enlarged blind spots, diffuse widespread defects, neurologic-like defects (including quadrant defects), normal pattern, and others.^14^^,^^19^^,^^21^^,^^24^ In the original IIHTT, the most common VF defect was a partial arcuate defect with an enlarged blind spot, which was a notable feature of several of the 14 IIH-specific ATs identified via AA.^21^ We also applied AA to a dataset of VFs taken from healthy “control” eyes, resulting in the generation of 12 ATs that reflected normal vision, and which were notably distinct from the IIH ATs. In addition, decomposition of control VFs into the IIH ATs enabled us to establish a threshold AT weight of 9% as representative of relevant or meaningful weight change, distinct from normal fluctuations. Collectively, these findings suggest that these IIH ATs are indeed disease-specific and further support the assertion that AA can identify relevant patterns of VF loss, as has been done in glaucoma.

Expert clinician classification and monitoring of VF defects is performed using all the perceived content in the visual field display. In addition, the detection and monitoring of different types of regional VF defects often relies on individual clinician interpretation, which is qualitative and can vary based on the interpreter. By contrast, AA decomposes VFs into multiple archetypes, each with an associated weighting coefficient. This allows more subtle patterns to be uncovered, even when applying a conservative rule of including only relevant (≥9% weight) ATs to analyze a specific VF. AA thus allows potentially important patterns of VF dysfunction to be seen rather than just selecting the major pattern features and provides quantitative measurement of each pattern's representation within the entire VF.

Furthermore, global VF indices such as MD and PSD may fail to fully convey regional changes in VF function. This may occur, in part because although MD is calculated from all of the total deviation points, it is weighted to the points with less variance, which are the most central points and biases against the more variable peripheral locations in the visual field. The PSD is a pattern-based index representing the extent of variation within a VF. While eyes with normal vision will display MD values closer to zero, *both* those with either normal or very poor vision can have PSD values closer to zero.^25^ We suggest that AA provides an additional method of assessing the variation in deficits within a VF. Within our 14-AT model, the strong correlation between AT1 weight and MD at baseline was expected, as AT1 represents a normal VF. As AT1 weight increases, the VF normalizes, and MD increases concurrently. The moderate correlation between AT Sum scores and MD at baseline was also expected, given that the AT Sum score provides an overall representation of AA-determined VF dysfunction at a single time point. However, the AT sum score correlated only mildly with PSD. For each AT sum score, we observed a wide range of associated MD and PSD values. This was not unexpected because PSD may be close to zero when the VF is either normal or severely compromised. However, there is considerable variance in IIH VF function that is unexplained by MD or PSD alone, because VFs with comparable MD and PSD values may carry different AT weighting coefficients (Fig. 7).

AA provides a new, quantitative method of identifying component regional VF defects in eyes of IIH patients with mild VF loss. Prior descriptive interpretations appear incomplete and may be more fully identified and quantified by AA for both research and clinical purposes. In our future studies of outcome data from the IIHTT, we will be exploring the question of whether AT weights at baseline or early in the treatment course may predict visual outcome, treatment effect, or treatment failure.

## Conclusion

Archetypal analysis is well suited for the evaluation of visual fields in IIH, an optic nerve disorder that can be improved with therapy. AA identifies IIH-specific, quantifiable patterns of VF loss in this disease. AA may thus prove useful in the quantitative monitoring of VF defects over time. Although these results are encouraging, our analysis was only performed in eyes with mild VF damage (as was characteristic of those enrolled in the IIHTT). It is possible that additional patterns of visual field loss will be found, or the relative weights of each AT will differ for eyes with more severe IIH. Further research will involve application of this 14-AT model to real-world IIH datasets, with the inclusion of patients with worse visual field loss. Additional investigations may reveal specific VF features that are predictive of the VF outcome or reflect changes to facilitate monitoring.

## Supplementary Material

Supplement 1
