# Supplementary material for: Unsupervised Machine Learning Identifies Quantifiable Patterns of Visual Field Loss in Idiopathic Intracranial Hypertension
Source: Transl Vis Sci Technol. 2021 Aug 30;10(9):37. doi: 10.1167/tvst.10.9.37 (PMC8411857; doi:10.1167/tvst.10.9.37)
Supplement: Supplement 1 [file tvst-10-9-37_s001.docx]

**Supplemental Figures and Tables**

**
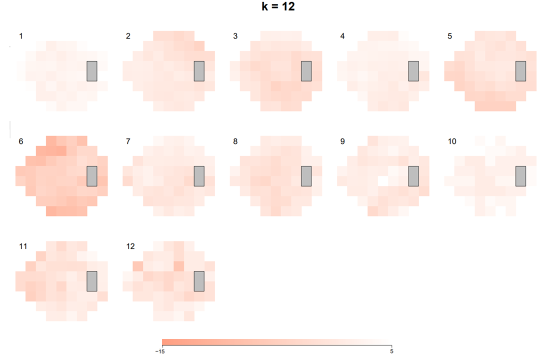
**

**Supplemental Figure 1:** Map outlining the 12 archetypes (ATs) contained within our model for the control visual field (VF) dataset from normal, healthy eyes. The scale at the bottom denotes the total deviation (TD) values associated with the different shades of red seen within each AT pattern. Note this scale has a shorter interval compared with the AT map shown in Figure 2 which describes idiopathic intracranial hypertension (IIH) eyes, as the control eye VFs had TD values within a more narrow and less negative range (-15 to 5 dB for control eyes, as opposed to -35 to 5 dB for IIH eyes).

**
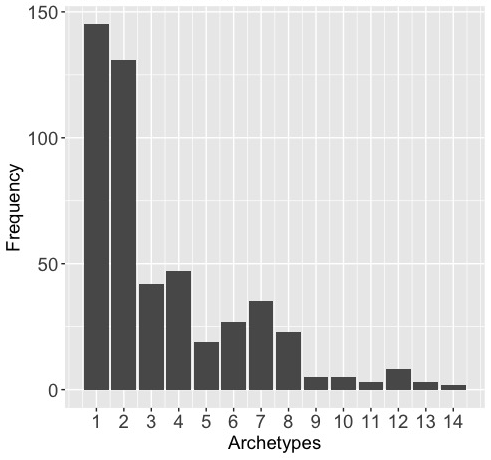
**

**Supplemental Figure 2:** Frequency of non-study eyes with archetype weight ≥ 9% at baseline (for each archetype as listed). Note that although these eyes had papilledema, most eyes were normal or near normal.

| Archetype | Mean Weight at Baseline | 95% CI Lower Bound | 95% CI Upper Bound |
| --- | --- | --- | --- |
| AT1 | 0.147 | 0.126 | 0.168 |
| AT2 | 0.239 | 0.209 | 0.270 |
| AT3 | 0.101 | 0.081 | 0.120 |
| AT4 | 0.077 | 0.057 | 0.097 |
| AT5 | 0.070 | 0.050 | 0.090 |
| AT6 | 0.067 | 0.049 | 0.084 |
| AT7 | 0.076 | 0.056 | 0.096 |
| AT8 | 0.059 | 0.040 | 0.078 |
| AT9 | 0.036 | 0.025 | 0.047 |
| AT10 | 0.029 | 0.025 | 0.034 |
| AT11 | 0.022 | 0.018 | 0.026 |
| AT12 | 0.032 | 0.025 | 0.039 |
| AT13 | 0.027 | 0.021 | 0.034 |
| AT14 | 0.018 | 0.014 | 0.022 |

**Supplemental Table 1: Baseline Mean Archetype Weight Distribution.** Includes data for all study eyes. CI = confidence interval.

| Archetype | r | P-value |
| --- | --- | --- |
| AT1 | 0.693 | < 0.001 |
| AT4 | 0.157 | 0.043 |
| AT7 | 0.162 | 0.038 |
| AT12 | -0.188 | 0.015 |
| AT13 | -0.350 | < 0.001 |
| AT14 | -0.268 | < 0.001 |

**Supplemental Table 2:** **Mean Deviation and Archetype Weight Correlations.** Spearman correlation coefficients between each archetype weight and mean deviation at baseline are shown with their corresponding p-values (only significant correlations shown).

| Archetype | r | P-value |
| --- | --- | --- |
| AT1 | -0.338 | < 0.001 |
| AT2 | -0.675 | < 0.001 |
| AT10 | -0.195 | 0.012 |
| AT11 | -0.432 | < 0.001 |

**Supplemental Table 3:** **Pattern Standard Deviation and Archetype Weight Correlations.** Spearman correlation coefficients between each archetype weight and pattern standard deviation at baseline are displayed with their corresponding p-values (only significant correlations shown).

| Archetype | r | P-value |
| --- | --- | --- |
| AT1 | -0.19 | 0.03 |
| AT2 | -0.21 | 0.01 |
| AT3 | -0.27 | 0.002 |
| AT4 | 0.22 | 0.009 |
| AT7 | 0.37 | < 0.001 |
| AT8 | 0.37 | < 0.001 |
| AT12 | 0.19 | 0.03 |
| AT13 | -0.29 | < 0.001 |

**Supplemental Table 4: Frisén Grade and Archetype Weight Correlations.** Spearman correlation coefficients between each archetype weight and Frisén grade at baseline are displayed with their corresponding p-values (only significant correlations shown). AT7 and AT8 are abnormal patterns due to an enlarged blind spot.

| **Archetype** | **Study Eyes with a Dominant AT (N=93)** | **Full Match (N=65)** | **Partial Match**  **(N=2)** | **Full or Partial Match Total (N=67)** | **Full Match** | **Partial Match** |
| --- | --- | --- | --- | --- | --- | --- |
| AT1 | 6 | 2 | 2 | 4 | Normal | N/A |
| AT2 | 38 | 14 | 0 | 14 | Mild widespread loss | N/A |
| AT3 | 7 | 7 | 0 | 7 | Superior partial arcuate | Superior depression, other NFB (+/- EBS) |
| AT4 | 7 | 7 | 0 | 7 | EBS | EBS with NFB |
| AT5 | 9 | 9 | 0 | 9 | Inferior nasal step or NFB | EBS with NFB |
| AT6 | 4 | 4 | 0 | 4 | Superior nasal step or NFB | EBS with NFB |
| AT7 | 13 | 13 | 0 | 13 | EBS | EBS with NFB |
| AT8 | 7 | 7 | 0 | 7 | EBS | EBS with NFB |
| AT9 | 2 | 2 | 0 | 2 | Superior temporal quadrantanopia | Superior temporal quadrantanopia +/- EBS |
| AT10 | 0 | 0 | 0 | 0 | Inferior NFB | N/A |
| AT11 | 0 | 0 | 0 | 0 | Paracentral field loss | N/A |
| AT12 | 0 | 0 | 0 | 0 | Inferior temporal quadrant defect | N/A |
| AT13 | 0 | 0 | 0 | 0 | Peripheral rim or partial peripheral rim | N/A |
| AT14 | 0 | 0 | 0 | 0 | Widespread loss | N/A |

**Supplemental Table 5:** **Matching between Dominant ATs and Expert Classifications.** This table displays the number of baseline VFs with a dominant AT (> 50%) that had a full match or partial match with expert classifications from the idiopathic intracranial hypertension treatment trial. The two right side columns show criteria used for matching (EBS = enlarged blind spot, NFB = nerve fiber bundle.
